# Molecular Docking Studies of Phytocompounds from the Phyllanthus Species as Potential Chronic Pain Modulators

**DOI:** 10.3797/scipharm.1408-10

**Published:** 2014-11-08

**Authors:** Atul R. Chopade, Fahim J. Sayyad, Yogesh V. Pore

**Affiliations:** 1 Department of Pharmacology and Pharmacognosy, Government College of Pharmacy, Karad, District Satara, 415124 Maharashtra, India; 2 Department of Pharmacology, Rajarambapu College of Pharmacy, Kasegaon, District-Sangli, 415404 Maharashtra, India; 3 Department of Pharmaceutical chemistry, Government College of Pharmacy, Karad, District Satara, 415124 Maharashtra, India

**Keywords:** InflammatoryPain, Docking, Phytochemical ligands, Phyllanthus

## Abstract

The study of inflammatory pain has been one of the most rapidly advancing and expanding areas of pain research in recent years. Studies from our lab have demonstrated the chronic pain-modulating potential of the Phyllanthus species and their probable interaction with various inflammatory mediators involving enzymes like COX-2 and PGE synthase, cytokines like TNF-alpha and IL-1 beta, and with the NMDA receptor. Inflammatory mediators which play a crucial role in chronic inflammatory hyperalgesia and its subsequent modulation were selected for their interactions with 86 structurally diverse phytoconstituents identified from the Phyllanthus species.

The docking analysis of the target proteins with the phytochemical ligands was performed using VLifeMDS software. The docking scores and analysis of the interactions of the phytocompounds with target proteins suggest that important molecules like lupeol, phyllanthin, hypopyllanthin, corilagin, epicatechin, and most of the other compounds have the ability to bind to multiple targets involved in inflammatory hyperalgesia.

Our study strongly suggests that the findings of the present study could be exploited in the future for designing ligands in order to obtain novel molecules for the treatment and management of chronic pain.

## Introduction

The study of inflammatory pain has been one of the most rapidly advancing and expanding areas of pain research in recent years [1]. Inflammatory mediators are crucially involved in the genesis, persistence, and severity of pain following trauma, infection, or nerve injury. Studies have characterized the series of receptors, ion channels, and transmitters that are involved in the processing of inflammatory pain [2, 3]. Current research is focused on the key mechanisms that produce hyperalgesia that accompany inflammation [1–3]. There is a largely unmet medical need for the treatment of inflammatory pain initiated by tissue damage or inflammation that manifests as spontaneous pain and pain hypersensitivity (hyperalgesia) [1, 4]. Also, the inflammatory mediators that interact with neurons to produce hyperalgesia are being explored.

With the primary aim to explore novel, leading compounds for the treatment of inflammatory hyperalgesia, we planned to focus our research on natural products and phytochemicals. Many pharmacological classes of drugs include a natural product prototype [5, 6]. Aspirin, atropine, artimesinin, colchicine, ephedrine, physostigmine, pilocarpine, quinine, quinidine, reserpine, taxol, vincristine, and vinblastine are a few examples of important molecules that medicinal plants have given us in the past. Also, there are many historical examples in which the natural product has not just been the medicinal product, but has also helped in revealing novel aspects of pharmacology and physiology [5–7]. For example, morphine pointed the way to the receptors affected by endogenous opioids; muscarine, nicotine, and tubocurarine helped explore the different types of acetylcholine receptors; digitalis from foxglove showed the role of sodium-potassium-ATPase, and so on.

The plants belonging to the genus Phyllanthus (Euphorbiaceae) are widely distributed throughout the world. A great variety of species of plants belonging to the genus Phyllanthus have been phytochemically and pharmacologically investigated and many molecules have been isolated and identified [8–12]. Phytochemical studies carried out on the Phyllanthus have revealed various classes of compounds, including alkaloids, flavonoids, lignans, phenols, and terpenes, which seem to be mainly responsible for the pharmacological actions reported in relation to these plants. Most of these compounds were found to interact with most key enzymes, such as aldose reductase, angiotensin converting enzyme, mitrochondrial ATPase, both cylo- and lipooxygenases, phospholipase A2, tyrosine kinase, reverse transcriptase, and phosphodiesterases [8–12]. Recently, we have also reported the diverse pharmacological activities of *P. amarus* and *P. fraternus* standardized extracts and their significant pain modulating potential [13–21]. The outcomes of the results from the studied *in vitro* and *in vivo* models are presented in a comparative manner in Table 1. Evidence from our studies of acute and chronic pain models suggests that Phyllanthus extracts are responsible for inhibiting important inflammatory pain mediators like prostaglandins, interleukins, TNF etc., so as to get a clue on exactly which class of compounds or phytocompounds were responsible for the observed activity. We thought of utilising docking analysis to predict the novel leads and to concentrate on a particular class of phytocompounds.

**Tab. 1:**
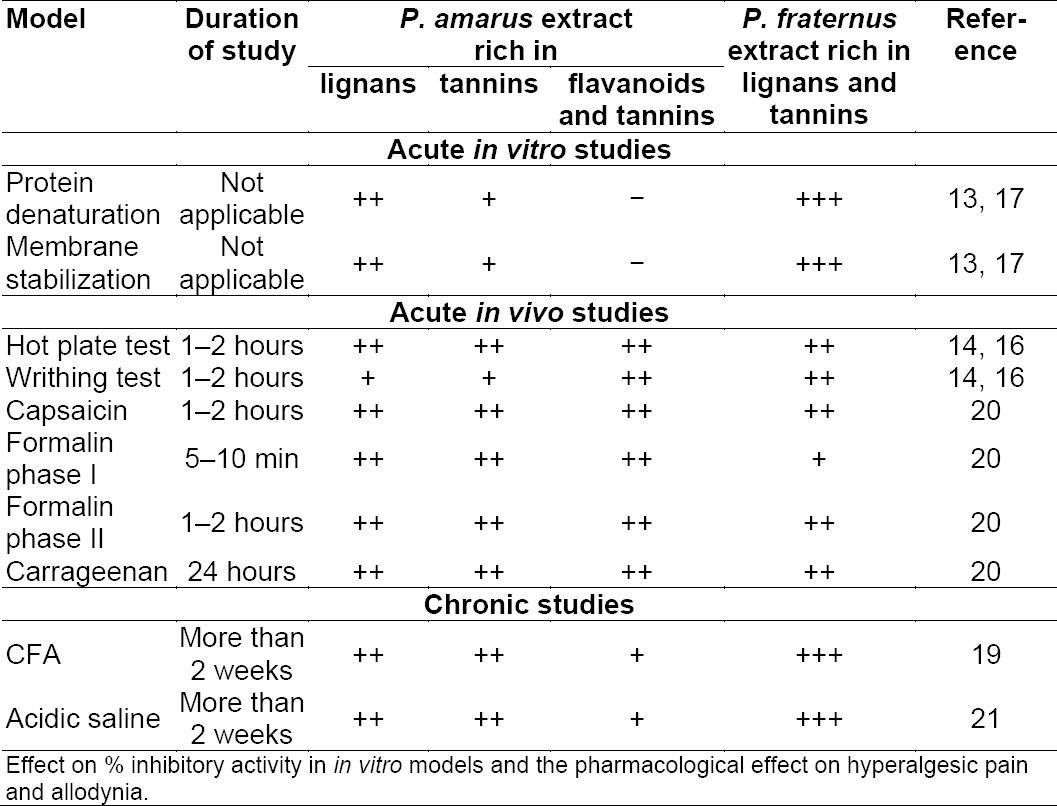
Comparison of the observed effects in the studied *in vitro* and *in vivo* models

The present study is also an extension of molecular docking analysis with an attempt to set a logical correlation for *in vitro* and *in vivo* outcomes with an *in silico* study. Eighty-six phytocompounds (ligands) were selected for docking analysis; their details are tabulated in Table 2.

**Tab. 2:**
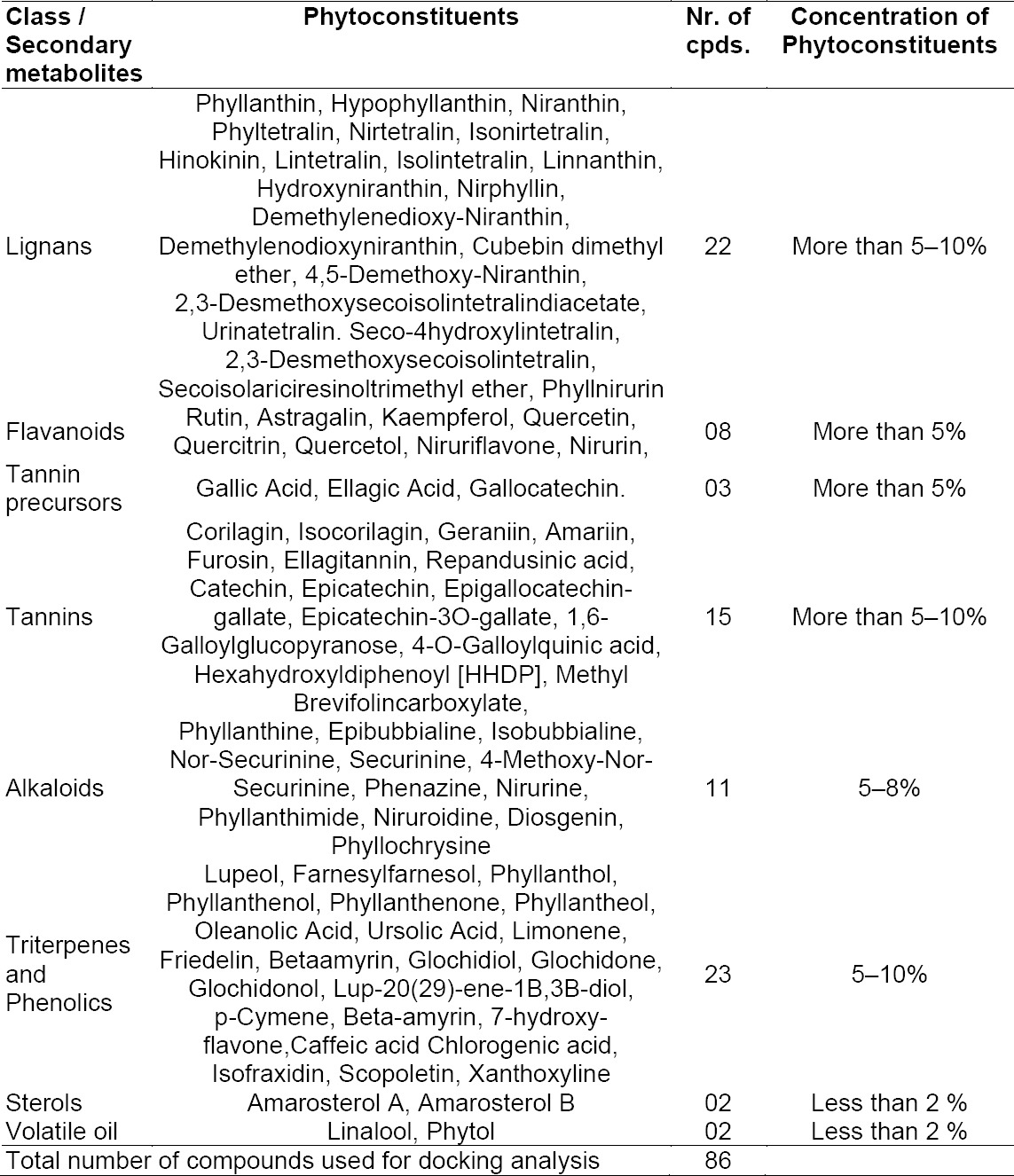
List of various classes of phytoconstituents and their reported concentrations from the Phyllanthus species included in the study [8–12]

To assist in determining the potential mechanisms of action of the phytochemical compounds from the Phyllanthus species, we carried out flexible docking analysis of structurally diverse phytocompounds isolated from the Phyllanthus species for their selective inhibitory activity against five targets (enzymes like COX-2 and PGE synthase, cytokines such as TNF-alpha and IL-1 beta, and the NMDA receptor) which play a crucial role in chronic inflammatory hyperalgesia and its subsequent modulation.

## Results

VLifeMDS provided a facility to dock different ligands in protein binding sites chosen by the user. VLifeMDS provided both rigid (no torsional flexibility for a protein as well as a ligand) and flexible (torsional flexibility to a ligand with a rigid protein) docking of the molecules. The target or receptor was either experimentally known or theoretically generated through knowledge-based protein modeling or homology modeling. The molecular docking tool has been developed to obtain a preferred geometry of interaction of ligand–receptor complexes having minimum interaction energy based on different scoring functions viz. only electrostatics, the sum of steric and electrostatic (parameters from the force field), and the dock score. This utility allowed us to screen a set of compounds for lead optimization. VLifeMDS uses the genetic algorithm, Piecewise Linear Pairwise Potential (PLP) and Grid algorithms to minimize the interaction energy between the ligand and receptor protein.

The downloaded protein databank file of the target protein was checked for any errors in the protein structure with yhe help of biopredicta tools. Incomplete residues or incomplete atoms were either mutated or edited with the help of adjacent residues. Unwanted chains of the protein were removed by selecting the chain and deleting it from the structure. The target protein was checked for crisscross residues, a local geometry check, and a Ramachandran plot with the help of Biopredicta tools and coordinates. For the local geometry check, the tools settings were kept as follows: the bond length was 20%, bond angle 20%, and bond length 10%. The protein was finally optimized by using the computed forcefield option to minimize errors between the protein-ligand interactions.

The structures of 89 phytocompounds (ligands) were drawn in 2D and converted into an optimized 3D form before using VLifeMDS computational software. The phytochemical ligands were docked with the five selected receptors. The ligands showed unique kinds of interactions with selected receptor proteins in the present study. The protein-ligand interactions were observed during docking analysis, concentrating on the study of the docked poses which showed significant dock scores. The docking scores of most of the targets were fairly better as compared to the co-crystal ligand scores, which indicate better binding of the compounds as compared to the co-crystal ligands. The molecular docking scores identified the ligands that bind with similar orientation as observed from the reference ligands. Most of the phytocompounds (ligands) made good docking poses in comparison to the reference ligand. Selective ligands docked deeply within the binding pocket region, suggesting their complementary shape with the reference ligands. The Pi stacking, H-bonding, and hydrophobic interactions of the ligands with receptor proteins were analyzed which revealed a novel set of information. The results of the docking analysis and the interactions with the selected receptor proteins are discussed in the following sections.

### Docking Studies of Phyllanthus Ligands with Cyclooxygenases

The results of the interactions of ligands (Phyllanthus compounds) with the COX receptor are summarized in Table 3. The ligands show a unique set of interactions viz. Pi stacking, H-bonding, and hydrophobic. The docked ligands show scores ranging from −38 to −119, with the best-scored ligand being lupeol. The best docking pose of lupeol and the co-crystal ligand are as shown in the figure below. The best scoring pose of lupeol did not show Pi stacking or any H-bonding, but they showed specific hydrophobic interactions.

**Tab. 3:**
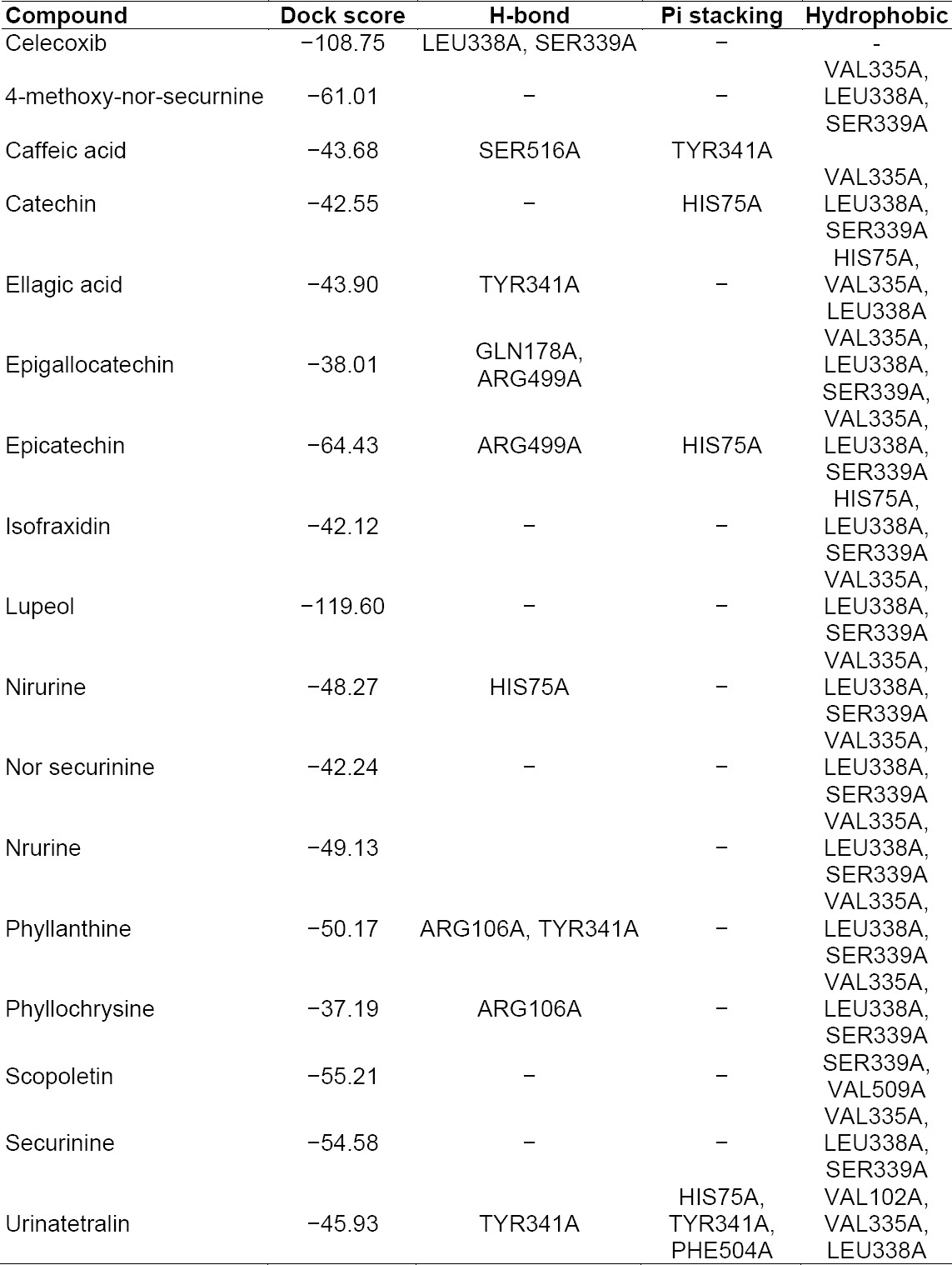
Summary of docking analysis of the COX receptor with Phyllanthus compounds

**Fig. 1:**
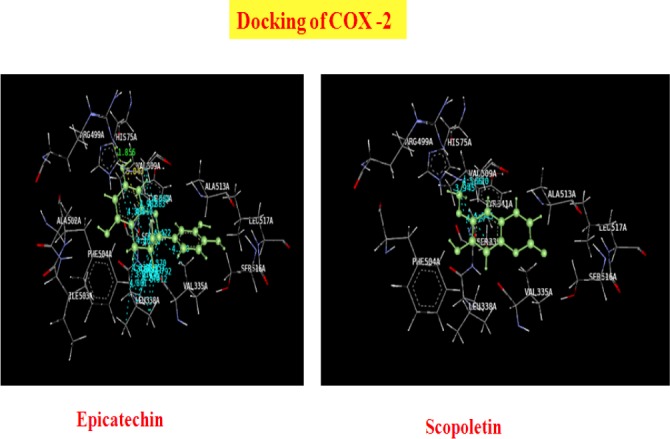
Best docking of poses of Epicatechin and Scopoletin with COX-2

The second best ligand epicatechin with a dock score of −64.43 showed H-bond interactions with ARG499A, Pi stacking interactions with HIS75A, and specific hydrophobic interactions. The 3D presentation of the best-docked pose of epicatechin and scopoletin with COX-2 are represented in Figure 1.

### Docking Analysis of PGE Synthase with Phyllanthus Compounds

**Fig. 2:**
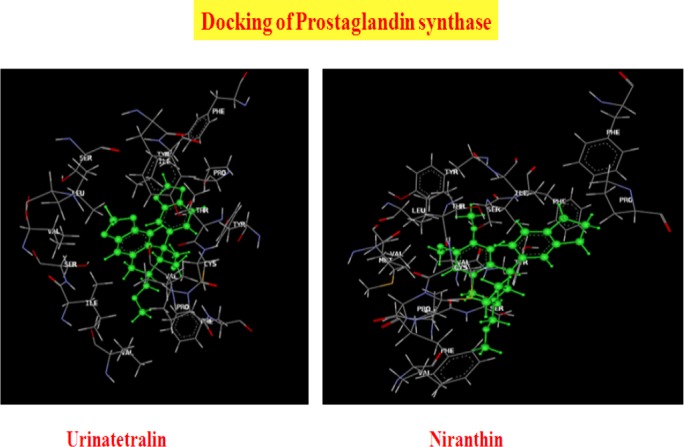
Best docking of poses of Urinatetralin and Niranthin with PGE

Thirty-six phytocompounds showed good affinity for PGE synthase. The docked ligands showed scores ranging from −35 to −88, with the best-scored ligand being lupeol. The best-scoring pose of lupeol did not show Pi stacking and H-bonding interactions. The next best ligands, urinatetralin and niranthin, with dock scores of −71.02 and −54.22, respectively, did not show Pi stacking nor H-bonding interactions, but rather unique hydrophobic interactions. An interesting ligand, lintetralin, with a dock score of −36.54, showed H-bonding with a lys269 residue, Pi stacking interactions with the tyr107a and tyr107a residues, and characteristics of hydrophobic interactions. The results and outcomes of the docking analysis and interactions of ligands (Phyllanthus compounds) with PGE synthase is summarized in Table 4. The 3D presentation of the best-docked pose of urinatetralin and niranthin with PGE synthase are represented in Figure 2.

**Tab. 4:**
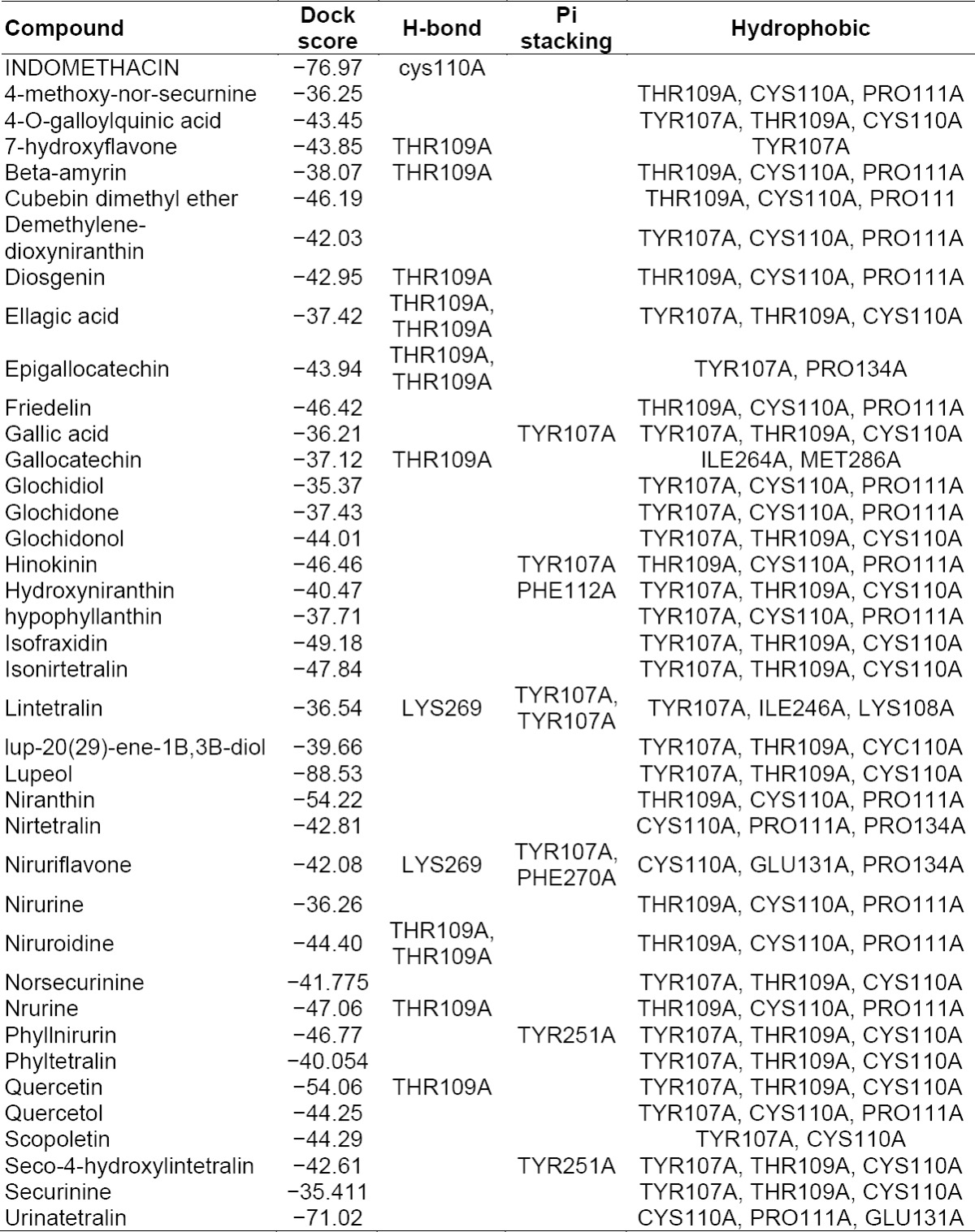
Summary of docking analysis of PGE synthase with Phyllanthus compounds

### Docking Analysis of TNF-Alpha with Phyllanthus Compounds

**Fig. 3:**
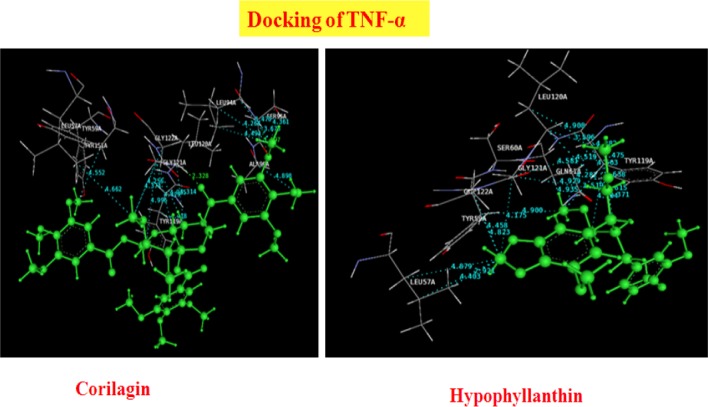
Best docking of poses of Corilagin and Hypophyllanthin with TNF-α

Thirty phytocompounds of the Phyllanthus species have an affinity for TNF-alpha. Most of the compounds showed good dock scores as compared with the standard reference ligand in the present study. The dock scores observed in the present study range from −35 to −67, with the best-scored ligand being lupeol. The best-scoring pose of lupeol with a dock score of −67.91 did not show Pi stacking nor H-bonding interactions, but rather unique hydrophobic interactions. The second best ligand quercetol, with a dock score of -52.76, showed no H-bond, but instead Pi stacking with the tyr119A and tyr119A residues, and hydrophobic interactions with pro117A, pro117A, and tyr119A. An interesting ligand, quercetin with a dock score of −41.95, showed H-bonding interactions with the tyr119A residue, Pi stacking interactions with the tyr119A and tyr119A residues, and characteristics of hydrophobic interactions. The results of the docking analysis and interactions of ligands (Phyllanthus compounds) with TNF-alpha are tabulated in Table 5. The 3D presentation of the best-docked pose of corilagin and hypophyllanthin with TNF-α is represented in Figure 3.

**Tab. 5:**
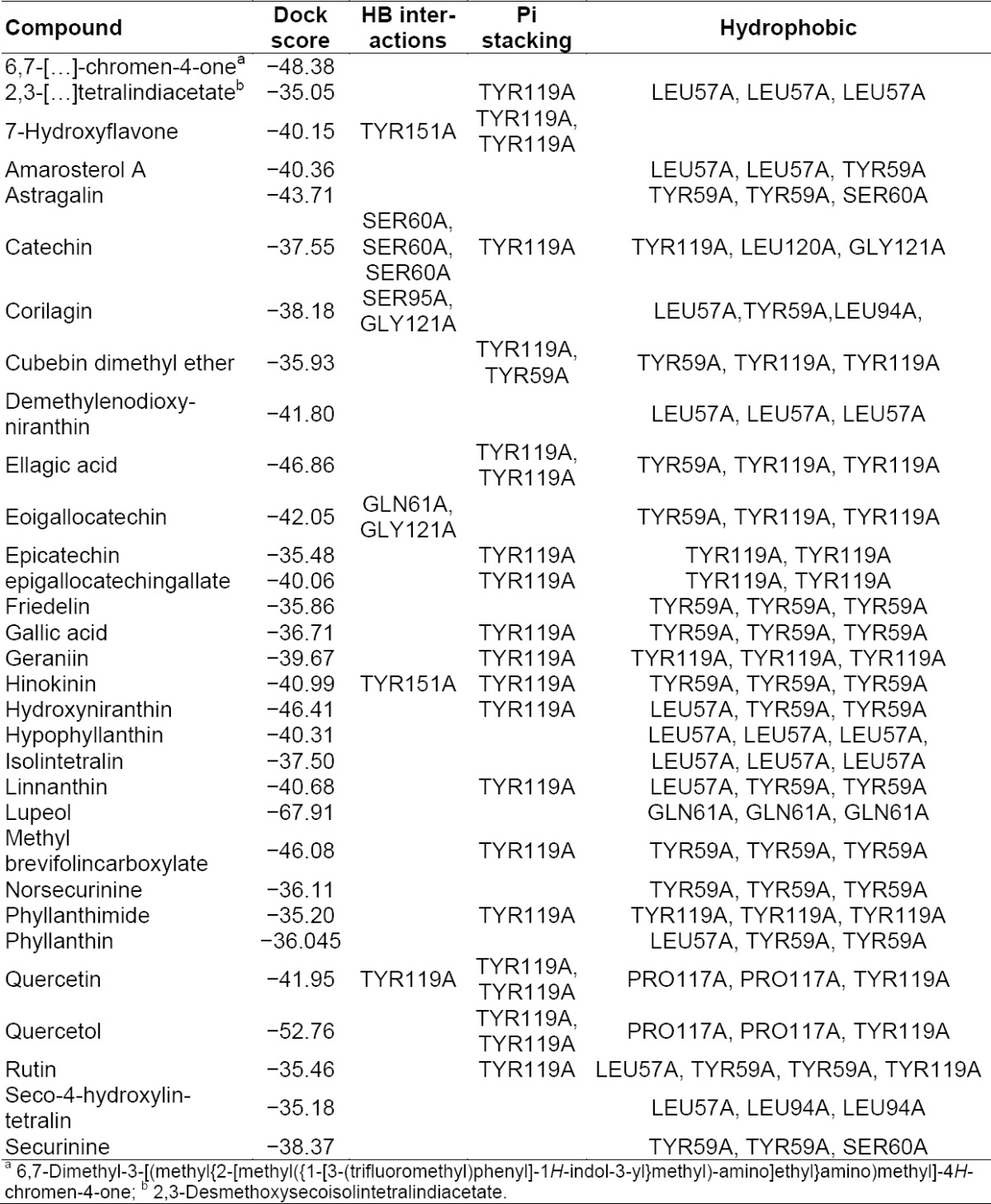
Summary of docking analysis of TNF-alpha with Phyllanthus compounds

### Docking Analysis of IL-beta with Phyllanthus Compounds

**Fig. 4:**
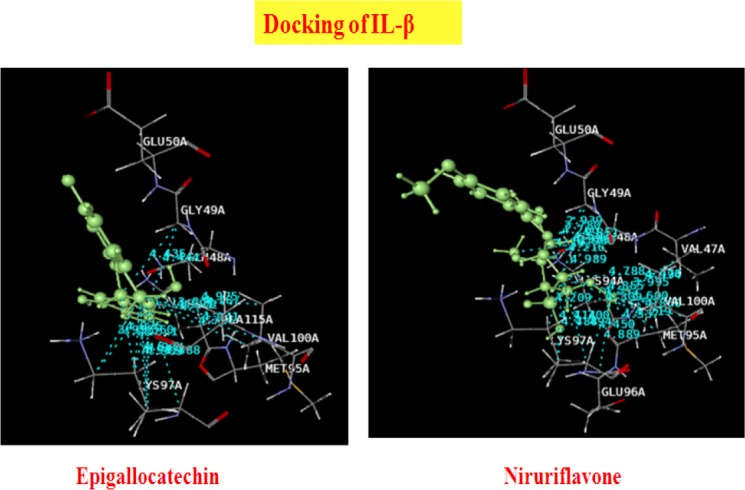
Best docking of poses of Epigallocatechin and Niruriflavone with IL-β

The results and outcomes of the docking analysis and interactions of ligands (Phyllanthus compounds) with IL-beta is summarized in Table 6. Thirteen phytocompounds of the Phyllanthus species have an affinity for IL-beta and none of these showed Pi stacking interactions. The docked ligands showed scores ranging from −32 to −59, with the best-scored ligand being lupeol. The best-scoring pose of lupeol did not show H-bonding interactions, but rather unique hydrophobic interactions. The second best ligand epigallocatechin, with a dock score of -50.16, showed H-bonding interactions with GLU50A and hydrophobic interactions with GLY49A, LYS97A, and ALA115A. Norsecurinine and phyllochrysine also showed H-bonding interactions with GLU50A. The 3D presentation of the best-docked pose of epigallocatechin and niruriflavone with IL-β are represented in Figure 4.

**Tab. 6:**
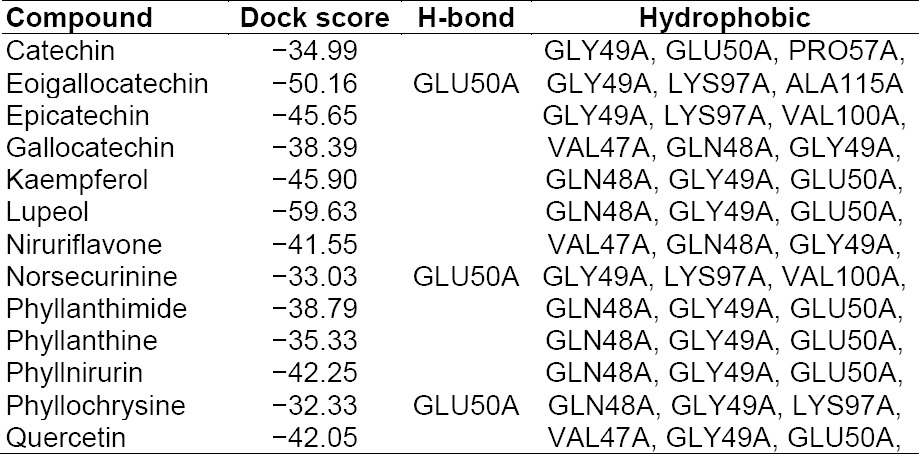
Summary of docking analysis of IL-BETA with Phyllanthus compounds

### Docking Studies of Phyllanthus Ligands with NMDA

**Fig. 5:**
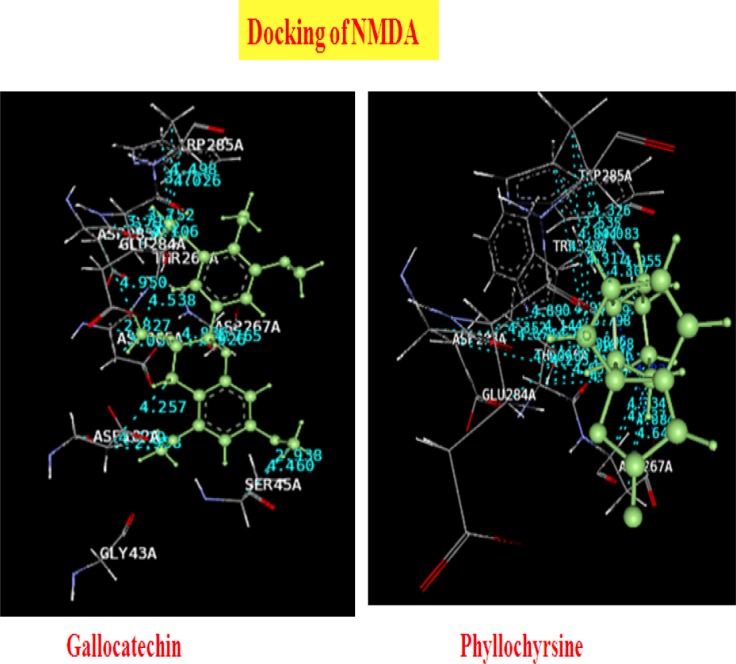
Best docking of poses of Gallocatechin and Phyllochrysine with NMDA

The results and outcomes of the docking analysis and interactions of ligands (Phyllanthus compounds) with the NMDA receptor are summarized in Table 7. Only six molecular structures of phytocompounds have affinity for the NMDA receptor. Epigallocatechin, gallocatechin, lupeol, norsecurinine, phyllochrysine, and scopoletin showed good docking scores. The results suggest these compounds are potent NMDA receptor antagonists. The docked ligands show scores ranging from −12 to −40, with the best-scored ligand being lupeol. The best docking pose of lupeol and the co-crystal ligand are as shown in the figure below. The best scoring pose of lupeol shows interesting hydrophobic interactions. The ligands were docked in the binding site of the NMDA receptor using the Phyllanthus co-crystal ligands as a reference for docking. The ligands did not show Pi stacking nor H-bonding interactions, except for scopoletin whose Pi stacking interaction was with TRP285A, but all the ligands showed significant hydrophobic interactions with a variety of residues. The 3D presentation of the best-docked pose of gallocatechin and phyllochrysine with NMDA are represented in Figure 5.

**Tab. 7:**
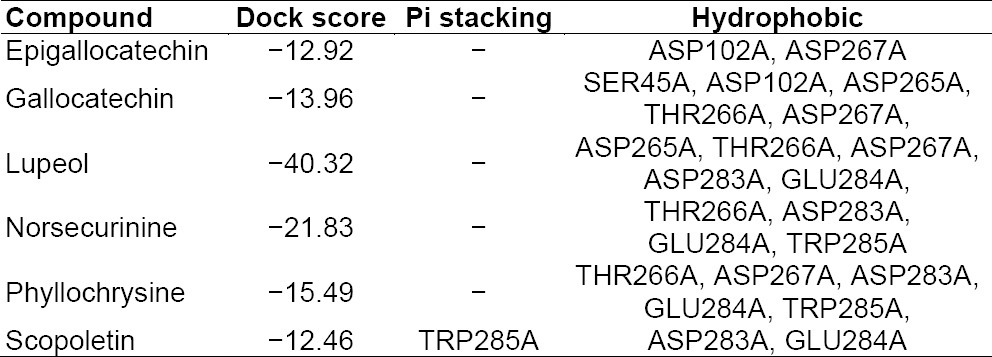
Summary of docking analysis of NMDA receptor with Phyllanthus compounds

## Discussion

In appreciating the inflammatory process, it is important to understand the role of chemical mediators that tend to direct the inflammatory response [1, 2]. Chemical mediators bind to specific receptors on target cells and can increase vascular permeability and neutrophil chemotaxis, stimulate smooth muscle contraction, have direct enzymatic activity, induce pain, or mediate oxidative damage [1, 2]. *In silico* molecular docking is one of the most powerful techniques to discover novel ligands for proteins of known structure and thus plays a key role in structure-based drug design. The *in vitro* and *in vivo* analysis carried out by us showed good results with regards to the anti-inflammatory potential of the Phyllanthus species; for details see Table 1. The present study may act as supportive evidence that substantiates the analgesic and anti-inflammatory properties of the Phyllanthus species, which may be because of their inhibiting ability of various phytoconstituents with inflammatory mediators identified from them.

**Fig. 6:**
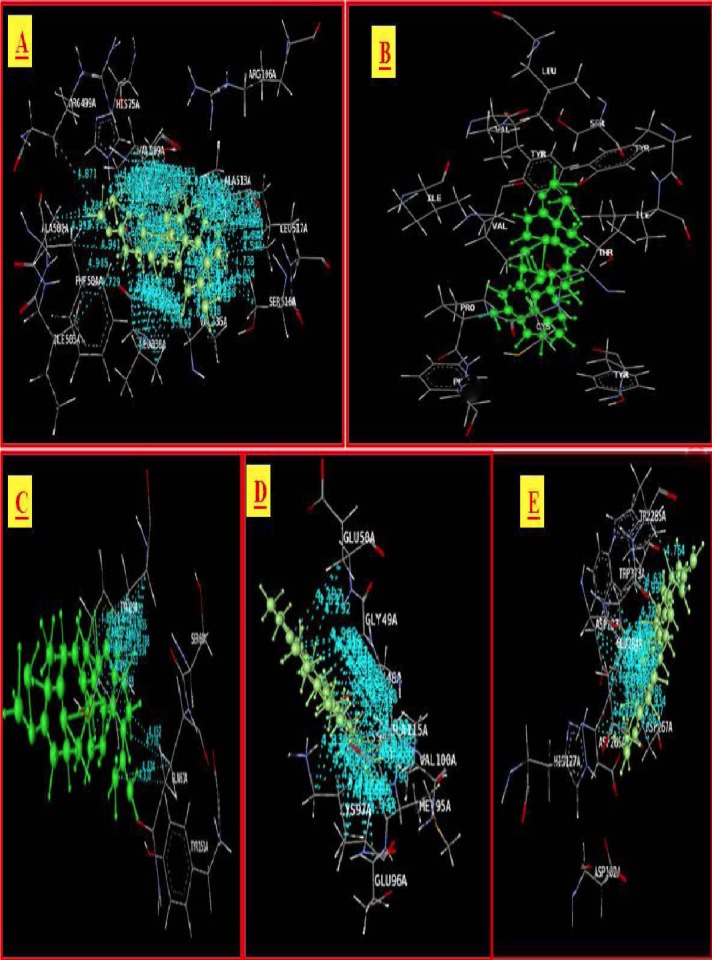
Best docking of poses of Lupeol with target proteins

The compound lupeol which is present in a small quantity in the Phyllanthus species has been included in the present docking analysis which shows highly significant dock scores and interesting interactions with all of the selected five targets. The interaction of lupeol with the target proteins viz. COX-2, PGE synthase, TNF-alpha, IL-1 beta, and NMDA has been depicted in Figure 6.

Phyto-pharmacological studies from our research lab suggest that the observed analgesic and anti-inflammatory activities with various standardized extracts of the Phyllanthus species were due to the presence of tannins, flavanoids, and lignans. There are a good number of reviews which report and confirm this above statement.

The HPTLC fingerprint analysis was carried out which was not been reported earlier. From the HPTLC chromatograms, we can interpret that the Phyllanthus extracts contained a considerable amount of lignans, flavonoids, and tannins. Therefore, the presence of these compounds might be the ultimate cause for their bioactivity. HPTLC analysis of Phyllanthus extracts revealed different peaks which were distinct for each extract.

As stated by the certificate of analysis of the sample providers, Phyllanthus amarus water extract [PAAE] contained 81.74% of water-soluble extractives determined by a gravimetric method. Phyllanthus amarus hydroalcoholic extract [PAHE] contained >5% corilagin (% w/w), Phyllanthus amarus methanol extract [PAME] contained >2.5% of phyllanthin and hypophyllanthin (% w/w),) determined by HPLC. Phyllanthus fraternus hydroethanolic extract [PFHEE] contained phyllanthin as well as corilagin. An HPTLC profile (chemical profile) of the Phyllanthus extracts also confirms and supplements the previous observation and strengthens the identification of Phyllanthus using the HPLTC profile in the present study.

**Fig. 7:**
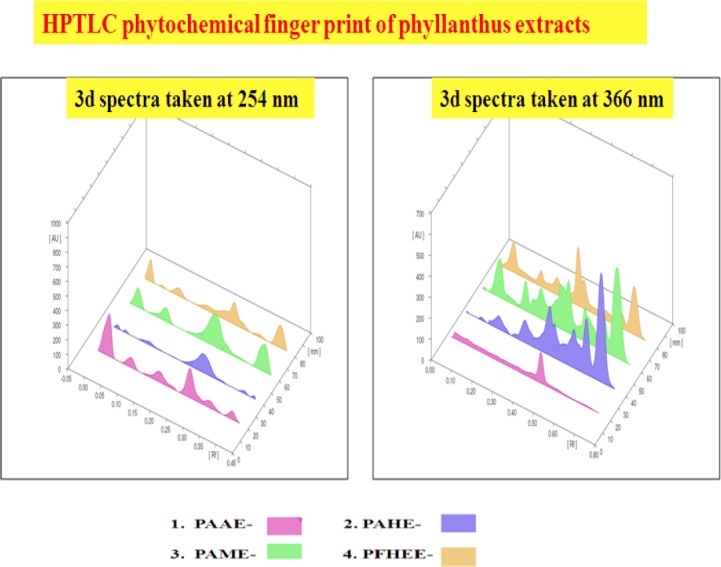
HPTLC phytochemical finger prints of Phyllanthus extracts. Phyllanthus amarus water extract [PAAE]; Phyllanthus amarus hydroalcoholic extract [PAHE]; Phyllanthus amarus methanol extract [PAME]; Phyllanthus fraternus hydro ethanolic extract [PFHEE].

The HPTLC fingerprint profile and 3D spectra of Phyllanthus extracts taken at 254 and 366 nm wavelengths are recorded in Figure 7. The phytochemical fingerprint analysis clearly indicates that the observed antihyperalgesic activity in the studied extracts can directly be assigned to the presence of lignans, tannins, and flavanoids.

In light of our *in vitro* and *in vivo* studies and as per review of literature we have discussed the results with reference to the compounds (ligands) belonging only to the lignans, tannins, and flavanoids class. Lignans are the major constituents present in the Phyllanthus extracts. They possess important pharmacological properties such as analgesic, antioxidant, anti-inflammatory, anti-arthritic, and immunomodulatory actions [22–24]. Purified lignans such as phyltetralin, nirtetralin, and niranthin isolated from it exhibit important *in vivo* and *in vitro* anti-inflammatory actions [22, 23]. Furthermore, the lignan-rich fraction and mainly niranthin were found to effectively interfere with the inflammatory response induced by platelet activating factor (PAF) [23]. While on the other side, tannins are also the major constituents present in the Phyllanthus extracts. The experimental data from the study by Moreira *et al.*, suggest that corilagin present in the Phyllanthus species shows anti-hyperalgesic activity that may be due to the interaction with the glutamatergic system [25]. It has been demonstrated that the anti-inflammatory actions of flavonoids *in vitro* or in cellular models involve the inhibition of the synthesis and activities of different pro-inflammatory mediators such as eicosanoids, cytokines, adhesion molecules, and C-reactive protein [26]. The flavonoids like rutin and quercetin have been described as cell-protecting agents because of their antioxidant, antinociceptive, and anti-inflammatory actions [27].

### COX Pathway

Inflammation causes the induction of cyclooxygenase-2 (COX-2), leading to the release of prostanoids, which sensitize peripheral nociceptor terminals and produce localized pain hypersensitivity. The COX inhibitors exert their analgesic effect by inhibiting the prostaglandin synthesis, thus reducing peripheral and central sensitization. In the various rodent models of carrageenan, CFA, zymosan, or formalin-evoked hyperalgesia, selective COX-2 inhibitors have markedly reduced the pain symptoms [28–32]. Similarly, we have also evaluated the Phyllanthus extracts in similar models [see Table 1] which also confirm the results of the docking analysis in the present study.

Recent evidence also indicates a role for COX inhibitors in the descending pain inhibition associated with PAG [periaqueductal gray region] as well [33]. The acidic saline-induced hyperalgesic model clearly suggests the role of the descending pain modulatory pathway associated with PAG [34–36]. Our study clearly indicates that Phyllanthus extracts significantly reduce the mechanical hyperalgesia following repeated intramuscular injections of acid and suggest that these can be useful in the treatment of chronic musculoskeletal pain syndromes such as fibromyalgia [21]. The tannins like ellagic acid, epicatechin, epigallocatechin, and urinatetralin, a lignan, showed good docking of COX-2 in the present study. Interestingly, alkaloids like phyllanthine, nirurine, nor-securinine, and Securinine also showed excellent docking scores and protein interactions.

### Prostaglandin Synthesis

Prostaglandins (PGs) have numerous and diverse biological effects on a variety of physiological and pathological events, such as the contraction of smooth muscle, inflammation, and blood clotting [37]. Out of the other types of prostaglandins, the PGE2 type plays a pivotal role in inflammatory hyperalgesia [38]. The crystal structure of N-terminal truncated mPGES-2 complexed with indomethacin, a significant non-steroidal anti-inflammatory drug, has been proposed [39, 40]. The crystal structure indicates that indomethacin inhibits both PGH2 synthesis and PGE2 synthesis. Evidence supporting the importance of PGE2 in the feedback loop comes from a previous study describing the induction of COX-2 expression by prostaglandins in human and mouse cell lines. For example, the results of the study by Pulichino *et al.*, suggest that the inhibition of PGE2 synthesis by NSAIDs and COX-2 inhibitors contributes to their efficacy in treating the signs of chronic inflammatory pain [40]. In our previous studies in the models of carrageenan, CFA, and formalin-evoked hyperalgesia, prostaglandins play an important role in evoking pain and hyperalgesia which the Phyllanthus extracts have shown good effects in the inhibition of hyperalgesia [19, 20]. In the present docking analysis, the lignans like hinokinin, hydroxyniranthin, hypophyllanthin, and urinatetralin showed good docking of microsomal prostaglandin E synthase type 2 (mPGES-2) in the present study. Tannins like ellagic acid, gallic acid, epicatechin, and epigallocatechin showed good docking of mPGES-2 in the present study. Interestingly, flavanoids like quercetin, quercetol, and niruriflavone showed excellent docking scores and protein interactions.

### Cytokine Pathways [Role of TNF-α and Interleukin-1β]

Cytokines play a fundamental role in the processes that cause inflammation, articular destruction, and the comorbidities associated with various chronic inflammatory diseases [41]. The two major cytokines involved in inflammatory pain and hyperalgesia are necrosis factors and the members of the interleukin family. In the present docking analysis, we have selected two highly significant cytokines such as TNF-α and Interleukin-1β in the current study.

#### TNF-α

Therapies targeting TNF-α are now also recognized to be effective in multiple other chronic inflammatory diseases, including juvenile RA (JRA), Crohn’s disease, psoriasis, psoriatic arthritis, and ankylosing spondylitis [41]. In the carrageenan-induced inflammation and chronic pain model of CFA, the concentration of TNF-α increases which is responsible for persistent pain and inflammation [19]. In the present docking analysis, the lignans like hinokinin, hydroxyniranthin, phyllanthin, hypophyllanthin, linnanthin, and urinatetralin showed excellent docking of the TNF-α receptor with characteristic protein interactions. Tannins like corilagin and geraniin showed high dock scores, while flavanoids like quercetin, quercetol, and rutin showed good dock scores and unique protein interactions.

#### Interleukin-1β

Interleukin-1β is a potent hyperalgesic agent, and its release can be induced, together with that of IL-6, by TNF-α–dependent and TNF-α-independent pathways [35, 41]. Interleukin-1β is the major cytokine stimulus for central COX-2 expression during inflammation [31]. Interleukin-1β stimulates IL-6 production during muscle injury, and the coordinated activities of both cytokines are necessary for repair and regeneration of muscle [22, 37]. Interleukin-1β can be expressed constitutively by myocytes and from resident macrophages [2, 36]. Targeting IL-1β and components of the receptor for IL-1β in various rodent models of arthritis is effective in reducing inflammation and particularly articular damage. In the carrageenan-induced inflammation and CFA model, the concentration of IL-1β increases with early injury and is responsible for the maintenance of chronic hypersensitivity. In the present docking analysis, it was observed that tannins and flavanoids from the Phyllanthus species have excellent affinity for IL-1β as compared with the other class of phytocompounds. Flavanoids like kaempferol, quercetin, and niruriflavone showed excellent dock scores and unique protein interactions. The tannins like epicatechin, epigallocatechin, and gallocatechin showed good inhibition of IL-1β in the present study.

### NMDA-Receptor Pathways

Different studies have shown the relationship between the increase in the antagonism of the NR2B subunit of ACC’s [anterior cingulate cortex] NMDA receptors and the level of tonic pain [42]. As an illustration, a study in mutant mice demonstrated that overexpression of the NR2B subunit of NMDA receptors in the ACC correlated with enhanced nociceptive responses in inflammatory pain models, such as in the formalin test and complete Freund’s adjuvant models [43, 44]. The formalin test showed enhanced second-phase pain response, and the CFA showed enhanced mechanical allodynia [23]. Moreover, another complementary study of the same group proved that tissue inflammation induces the upregulation of NR2B at the level of the ACC and enhanced the NMDA receptor-mediated response [43, 44]. Moreover, complementary studies with the pharmacological antagonism of NR2B at the level of the ACC (drugs Ro 25-6981 and Ro 63-1908) have shown a reduction of tonic pain in male and female rodents [45]. Our *in vivo* studies have also shown that Phyllanthus extracts have shown to inhibit the enhanced second-phase pain response in the formalin test and reverse the CFA-induced mechanical allodynia [19, 20]. Also, our study suggests a possibility that the inhibition of the glutamatergic system accounts for the antihyperalgesic effects observed in the model of chronic muscular pain. Tannins [like epigallocatechin and gallocatechin] and alkaloids [like norsecurinine and phyllochrysine] showed good docking affinity for the NR2B subunit of the NMDA receptor.

## Experimental

### Docking Tool and Algorithm

Molecular docking was completed using VLifeMDS version 4.3. The docking algorithm Biopredicta is based on a genetic algorithm which offers a successful strategy for globally searching the docked conformer’s space. Genetic algorithms allow a population of solutions to exist and in each ’generation,’ these can evolve by processes such as ’breeding’ and ’mutation’. Poor solutions are killed off, while good ones leave their offspring in future generations. Such algorithms may typically reach an excellent solution is a few tens of generations.

### Ligand Preparation [Structures of Compounds were Derived from Various Phyllanthus Species]

Eighty-six compounds were selected for the present experiment as listed in Table 1. The structures of various phytoconstituents reported in the Phyllanthus species were drawn in 2D and converted to 3D and were finally optimized for docking using VLifeMDS.

### Preparation of Enzyme Protein Structures [46–50]

Five popular targets (COX-2, PGE synthase, TNF-alpha, IL-1 beta, and NMDA) which play a crucial role in chronic pain and its subsequent modulation were selected for their interactions with the phytoconstituents isolated from various Phyllanthus species. The 3D structures of the enzyme proteins were downloaded from the Protein Data Bank (PDB). The targets used in the present study were COX-2 with PDBID-3LN1, PGE synthase with PDBID-1z9H, TNF-alpha with PDBID-2AZ5, IL-1 beta with PDBID-2NVH, and NMDA with PDBID-3JPW.

All the protein structures were subjected to a refinement and energy optimization before proceeding with the docking analysis. The cleaning of the proteins was performed by the addition of hydrogen atoms, completing incomplete residues. External ligands and ions of no significance present in the protein structure were deleted. The protein moiety was checked for crisscross residues, local geometry, and a Ramachandran plot using Biopredicta tools. For checking the local geometry, the allowed criteria were set to bond length 20%, bond angle 20%, and bond length 10%.

### Molecular Docking of Phyllanthus Compounds with Selected Targets

The rigid docking studies were performed using the PLP scoring function and the angle of rotation was set to 15 degrees. The molecular docking was performed for all the phytocompounds (ligands) from the Phyllanthus species with the best predicted poses of the interaction with the proteins under study. VLifeMDS provided a unique facility to dock different ligands in protein binding sites chosen by us. It also provided both rigid (no torsional flexibility for a protein as well as a ligand) and flexible (torsional flexibility to a ligand with a rigid protein) docking of the molecules.

### Analysis of Docked Protein-Ligand Complex Structures

Eighty-six optimized molecules were utilized to analyze and visualize the best molecularly docked poses. Before screening the ligands, the docking protocol was validated. The best orientations for the ligand-protein complexes were analysed. Distinction of good or bad docked conformation was based on the dock score. MDS used fitness functions on only electrostatic and both steric and electrostatic interactions between the receptor-ligand as well as the dock score scoring function. The Dock Score, or X-C score as it is called, computes the binding affinity of a given protein-ligand complex with a known 3D structure. The dock/X-C score scoring function include terms for van der Waals interactions, hydrogen bonding, a deformation penalty, and hydrophobic effects. The virtual screening technique employed in this study identified the ligands that bind in a comparable manner similar to the reference ligands (Celecoxib for COX-2, Indomethacin for PGE synthase, and 6,7-dimethyl-3-[(methyl{2-[methyl({1-[3-(trifluoromethyl)phenyl]-1*H*-indol-3-yl}methyl)amino]ethyl}amino)methyl]-4*H*-chromen-4-one for TNF-alpha) or the scores alone (for IL-1 beta and NMDA) where no reference ligands were available for docking.

## Conclusion

The present work was an attempt to computationally identify compounds which can bind to the crucial targets of chronic pain. The docking scores and analysis of the interactions of the compounds suggest that most of the compounds have the ability to bind to multiple targets involved in inflammatory hyperalgesia and it modulation. Experimental evaluation of the compounds like phyllanthin, hypophyllanthin, corilagin, etc., by logistic approaches would lead us to clinically effective molecules for treating various chronic pain disorders.
